# Population Genetic Variation in the Tree Fern *Alsophila spinulosa* (Cyatheaceae): Effects of Reproductive Strategy

**DOI:** 10.1371/journal.pone.0041780

**Published:** 2012-07-24

**Authors:** Ting Wang, Yingjuan Su, Yuan Li

**Affiliations:** 1 CAS Key Laboratory of Plant Germplasm Enhancement and Specialty Agriculture, Wuhan Botanical Garden, Chinese Academy of Sciences, Wuhan, Hubei, China; 2 State Key Laboratory of Biocontrol, School of Life Sciences, Sun Yat-sen University, Guangzhou, Guangdong, China; United States Department of Agriculture, United States of America

## Abstract

**Background:**

Essentially all ferns can perform both sexual and asexual reproduction. Their populations represent suitable study objects to test the population genetic effects of different reproductive systems. Using the diploid homosporous fern *Alsophila spinulosa* as an example species, the main purpose of this study was to assess the relative impact of sexual and asexual reproduction on the level and structure of population genetic variation.

**Methodology/Principal Findings:**

Inter-simple sequence repeats analysis was conducted on 140 individuals collected from seven populations (HSG, LCH, BPC, MPG, GX, LD, and ZHG) in China. Seventy-four polymorphic bands discriminated a total of 127 multilocus genotypes. Character compatibility analysis revealed that 50.0 to 70.0% of the genotypes had to be deleted in order to obtain a tree-like structure in the data set from populations HSG, LCH, MPG, BPC, GX, and LD; and there was a gradual decrease of conflict in the data set when genotypes with the highest incompatibility counts were successively deleted. In contrast, in population ZHG, only 33.3% of genotypes had to be removed to achieve complete compatibility in the data set, which showed a sharp decline in incompatibility upon the deletion of those genotypes. All populations examined possessed similar levels of genetic variation. Population ZHG was not found to be more differentiated than the other populations.

**Conclusions/Significance:**

Sexual recombination is the predominant source of genetic variation in most of the examined populations of *A. spinulosa*. However, somatic mutation contributes most to the genetic variation in population ZHG. This change of the primary mode of reproduction does not cause a significant difference in the population genetic composition. Character compatibility analysis represents an effective approach to separate the role of sexual and asexual components in shaping the genetic pattern of fern populations.

## Introduction

Almost all ferns are homosporous, producing only one type of spore that germinates to form a bisexual gametophyte [1]. In homosporous ferns, there are three possible modes of sexual reproduction [2]–[4]: (i) intragametophytic selfing - the union of sperm and egg from the same gametophyte resulting in a completely homozygous sporophyte; (ii) intergametophytic selfing - the union of sperm and egg from different gametophytes arising from spores from the same parental sporophyte (analogous to selfing in seed plants); and (iii) intergametophytic crossing - the union of sperm and egg from gametophytes arising from spores of different sporophytes (analogous to outcrossing in seed plants). In addition to these modes of sexual reproduction, many ferns also have the ability to propagate vegetatively in either the gametophyte or the sporophyte generation [5]–[7]. Numerous studies have been performed to evaluate the predominant mode of reproduction in natural populations of homosporous ferns [8]–[10].

**Table 1:** Primers used in ISSR analyses of *Alsophila spinulosa* (R  =  A, G; Y  =  C, T).

Primer	Sequence (5′–3′)	Primer	Sequence (5′–3′)
UBC815	CTC TCT CTC TCT CTC TG	UBC818	CAC ACA CAC ACA CAC AG
UBC820	GTG TGT GTG TGT GTG TC	UBC824	TCT CTC TCT CTC TCT CG
UBC825	ACA CAC ACA CAC ACA CT	UBC827	ACA CAC ACA CAC ACA CG
UBC835	AGAGAGAGAGAGAGA GYC	UBC840	GAG AGA GAG AGA GAG AYT
UBC841	GAG AGA GAG AGA GAGAYC	UBC844	CTC TCT CTC TCT CTC TRC
UBC846	CAC ACA CAC ACA CACART	UBC849	GTG TGT GTG TGT GTG TYA
UBC850	GTG TGT GTG TGT GTG TYC	UBC851	GTG TGT GTG TGT GTG TYG
UBC854	TCT CTC TCT CTC TCT CRG	UBC855	ACACAC ACA CAC ACA CYT
UBC864	TGT GTG TGT GTG TGT GRT	UBC866	CTC CTC CTC CTC CTC CTC
UBC873	GAC AGA CAG ACA GAC A	UBC880	GGA GAG GAG AGG AGA

Life-history traits, such as reproductive system and frequency of sexual versus asexual reproduction, are considered to be particularly important for shaping the level and structure of genetic variation in plant populations [11], [12]. In seed plants, it has been generalized that inbreeding potentially reduces within-population genetic variation but increases within-population genetic subdivision and among-population genetic differentiation, while outcrossing functions to enhance population genetic variation but impedes population subdivision and structuring [11], [13]. This generalization is upheld in some fern populations, such as populations of *Hemionitis palmata* [8], *Pleopeltis* species [14], *Polystichum otomasui* [15], *Asplenium trichomanes* subsp. *quadrivalens* [16], and *Dryopteris aemula* [17]. Yet notable exceptions do exist. For example, populations of *Botrychium virginianum* show little interpopulation genetic differentiation despite the possession of extremely high levels of within-population inbreeding [18], and populations of *Equisetum arvense* and *E. hyemale* exhibit considerable among-population divergence even though they reproduce primarily by outcrossing [19]. In the latter case, the high degree of differentiation was explained in terms of the life history of *Equisetum*, i.e. the inefficiency of spore germination and gametophyte reproduction in noncolonizing situations, which was considered to limit the gene flow between populations [19]. Asexual reproduction is generally hypothesized to decrease genetic variation (but depends on the number of genets) and promote genetic structure within and among populations [11], [20]. Nevertheless, such a hypothesis has also been challenged in some asexual angiosperms (e.g., *Taraxacum* agamosperms [21], [22]) as well as ferns (e.g., *Botrychium pumicola* [23]).

**Figure 1:**
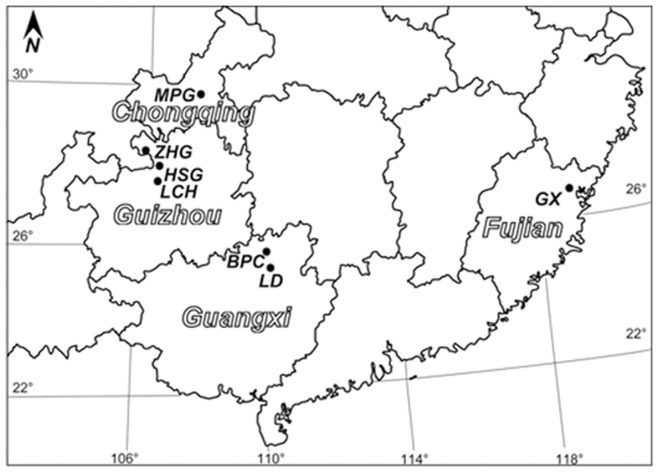
Locations of *Alsophila spinulosa* populations examined in this study.

*Alsophila spinulosa* (Hook.) Tryon (Cyatheaceae) is a diploid homosporous fern (2 n = 138) with a single, erect, tree-like rhizome up to 8 m tall [24]–[27]. In the Mesozoic Era, it was distributed worldwide, but much of its modern range is restricted to southern China (from 18.5° N to 30.5° N), southern Japan, and southeastern Asia after the Quaternary glaciations [28]–[30]. The species primarily lives in mountainous regions, occupying warm, humid, and shady habitats with acid soil (pH 4.5–5.5) at elevations of 400 to 900 m [28], [31]. Chiou et al. [32] reported that *A. spinulosa* is an outcrossing species, mainly producing sporophytes by intergametophytic crossing. In mainland China, natural populations of *A. spinulosa* have been recorded in Fujian, Guangdong, Hainan, Guangxi, Guizhou, Yunnan, Sichuan, and Chongqing [26], [28]. They are mostly small, isolated, and patchily distributed despite encompassing a wide geographic range. Thus, as early as in 1984, *A. spinulosa* was officially declared a first-grade protected plant by the State Environmental Protection Commission of China [33].

As a homosporous fern, *A. spinulosa* has the potential to maintain multiple reproductive modes and therefore affords a suitable study object to test the population genetic effects of different mating systems. The main purpose of the present study was to assess the relative contribution of sexual and asexual reproduction in shaping the population genetic composition by using inter-simple sequence repeat (ISSR) markers. In this respect, a novel molecular genetic approach, namely character compatibility analysis, has been shown to be very useful for separating the role of different modes of reproduction [34]–[36]. The approach is easy to apply to molecular markers such as ISSR because it is not hampered by dominance [35].

## Results

### Genetic Variation

Twenty ISSR primers (Table 1) were used to analyze 140 individuals collected from seven populations (Figure 1 and Table 2). They produced 102 bands, of which 74 (72.55%) were polymorphic. The number of bands amplified per primer varied from eight to 15 across populations, with an average of 8.71.

**Table 2:** List of code, location, and altitude of the seven *Alsophila spinulosa* populations included in this study.

Region	Population	Geographical coordinate	Altitude (m)
Guizhou	Liang Cha He (LCH), Chishui	106°01′E 28°25′N	470–557
	Hu Shi Gou (HSG), Chishui	106°01′E 28°29′N	555–602
	Zi Huang Gou (ZHG), Chishui	105°59′E 28°75′N	459–546
Guangxi	Ban Ping Cun (BPC), Longjiang	109°48′E 25°17′N	278–348
	Long Dou (LD), Longjiang	109°50′E 25°14′N	231–280
Chongqing	Mo Pan Gou (MPG), Fuling	107°26′E 29°41′N	437–477
Fujian	Gua Xi (GX), Fuan	119°31′E 26°58′N	204–570

Measures of genetic variation indicated that populations MPG, BPC, and GX might maintain higher levels of genetic variation than other populations (Table 3). Nevertheless, Mann-Whitney U tests revealed that none of these estimates of genetic variation differed significantly between the examined populations (P>0.5).

**Table 3:** Summary statistics revealed in seven populations of *Alsophila spinulosa* by using 20 ISSR primers.

Population	Number ofloci	Number of polymorphicloci	Percentage ofpolymorphic loci	Observed number of alleles	Effective numberof alleles	Nei’s gene diversity	Shannon’s index (95%confidence interval)
LCH	102	21	20.59%	1.2059	1.1602	0.0878	0.1627 (0.1525, 0.2927)
HSG	102	25	24.51%	1.2451	1.1790	0.1018	0.1487 (0.1546, 0.2834)
ZHG	101	27	26.73%	1.2673	1.1805	0.1046	0.1179 (0.1084, 0.2009)
BPC	100	34	34.00%	1.3400	1.2390	0.1336	0.1994 (0.1857, 0.3427)
LD	102	22	21.57%	1.2157	1.1532	0.0874	0.1531 (0.1341, 0.2885)
MPG	98	37	37.76%	1.3776	1.2705	0.1545	0.1453 (0.1422, 0.2611)
GX	102	36	35.29%	1.3529	1.2339	0.1382	0.1495 (0.1391, 0.2747)

Values of observed number of alleles and effective number of alleles are averages per locus.

### Identification of Genotypes

The polymorphic bands discriminated a total of 127 multilocus genotypes. The number of genotypes within each population varied from 15 to 20 (Table 4). In population HSG, no genotype was found more than once. Populations LCH, BPC, MPG, and GX each possessed only one genotype that occurred twice or three times; and population LD had two genotypes appearing twice. In population ZHG, however, there were three genotypes that were represented twice or three times. Additionally, no shared genotypes were detected among populations.

**Table 4:** Summary of ISSR genotype characters for each population.

Population	Number of fingerprinted individuals	Number of genotypes	Number of genotypes found once
HSG	20	20	20
LCH	20	18	17
ZHG	20	15	12
BPC	20	19	18
LD	20	18	16
MPG	20	18	17
GX	20	19	18
Total	140	127	118

### Population Genetic Differentiation

Pairwise *θ^B^* values and their 95% confidence intervals (CI) between the *Alsophila spinulosa* populations were estimated by running the *f*-free analysis in Hickory. The estimated *θ^B^* values between population pairs (excluding population ZHG) ranged from 0.1710 (CI: 0.0812–0.2935) to 0.3862 (CI: 0.2799–0.4968) (Table 5).

**Table 5:** Pairwise *θ^B^* values (above diagonal) and their 95% confidence intervals (below diagonal) between *Alsophila spinulosa* populations.

	LCH	HSG	ZHG	BPC	LD	MPG	GX
**LCH**		0.1710	0.2588	0.3836	0.3124	0.3120	0.3395
**HSG**	0.0812, 0.2935		0.1654	0.3862	0.2964	0.3060	0.3349
**ZHG**	0.1442, 0.3984	0.0808, 0.2854		0.3686	0.3235	0.3240	0.3115
**BPC**	0.2778, 0.4968	0.2799, 0.4968	0.2628, 0.4725		0.3169	0.3469	0.2782
**LD**	0.1949, 0.4505	0.1767, 0.4347	0.1987, 0.4624	0.2186, 0.4243		0.3256	0.2916
**MPG**	0.2018, 0.4390	0.1970, 0.4259	0.2125, 0.4461	0.2507, 0.4516	0.2178, 0.4432		0.2515
**GX**	0.2243, 0.4620	0.2237, 0.4541	0.1977, 0.4421	0.1882, 0.3771	0.1809, 0.4158	0.1541, 0.3692	

In the following sections, population ZHG will be shown to have a low level of matrix incompatibility and was dominant for asexual reproduction. Comparatively, the *θ^B^* values between population ZHG and the six other populations ranged from 0.1654 (CI: 0.0808–0.2854) to 0.3686 (CI: 0.2628–0.4725) (Table 5). Therefore, this population is no more differentiated than the other populations.

### Compatibility Analysis

**Figure 2:**
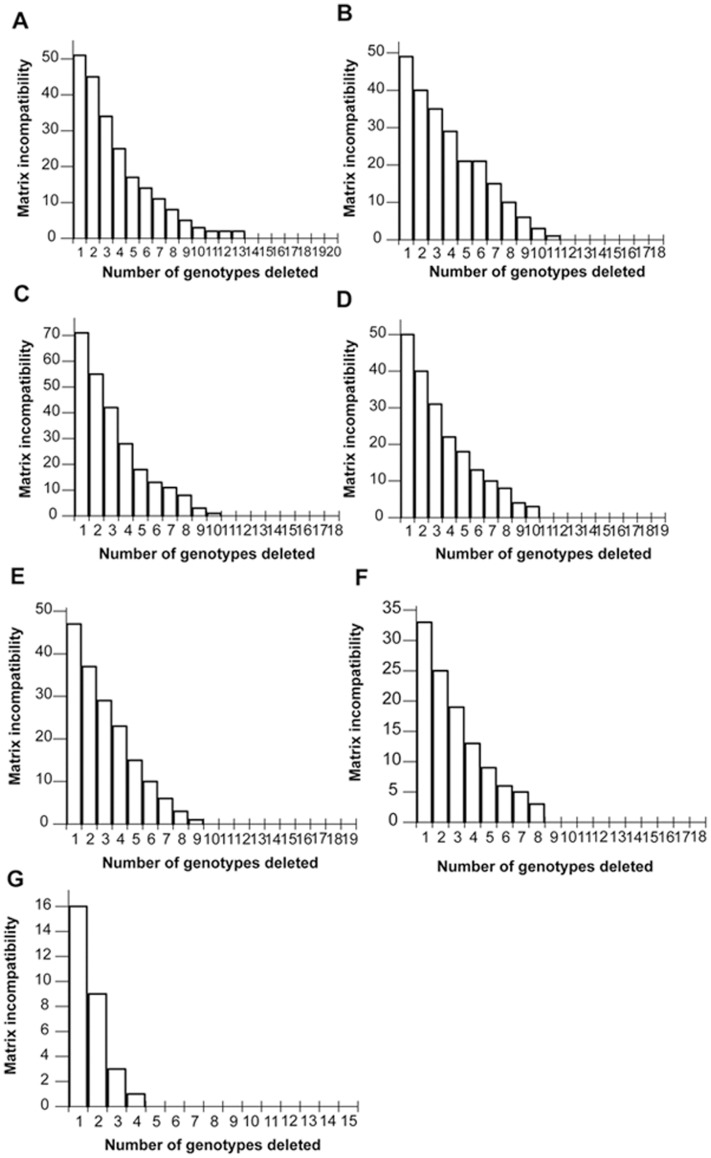
Matrix incompatibility upon successive deletion of genotypes with the highest number of incompatible character states. Bar diagrams A-G present results from populations HSG, LCH, MPG, BPC, GX, LD, and ZHG, respectively. Numbers on the horizontal axis refer to the number of genotypes that have been deleted sequentially.

All populations except ZHG displayed considerable matrix incompatibility. It took 14 of 20 (70.0%), 12 of 18 (66.7%), 11 of 18 (61.1%), 11 of 19 (57.9%), 10 of 19 (52.6%), and 9 of 18 (50.0%) genotypes to eliminate all the matrix incompatibility in the data of populations HSG, LCH, MPG, BPC, GX, and LD, respectively (Figure 2A–F). The continuous decrease of incompatibility showed that no major groups of genotypes were more incompatible with each other than with other individuals; namely, a minority of genotypes had strictly clonal relationships as well as a tree-like data structure in the populations. Here the tree-like data structure means that genotypes could be arranged in a phylogenetic tree without homoplasy [35]–[37]. In contrast, population ZHG possessed a much lower level of initial incompatibility, with only five of 15 (33.3%) genotypes needing to be deleted to achieve complete compatibility of the data set (Figure 2G). This sharp decline of matrix incompatibility count (MIC) suggested that there are only a few recombinant genotypes contributing to the incompatibility.

## Discussion

Molecular markers have been increasingly used to infer the influence of reproductive systems on patterns of population genetic variation in plants [36], [38], [39]. In this study, a more recently developed genetic approach, character compatibility analysis [35], was applied to evaluate the relative contribution of sexual recombination and somatic mutation to ISSR variations observed in the populations of *A. spinulosa*.

Analysis of character compatibility indicated that in six out of the seven *A. spinulosa* populations examined, sexual recombination had been the predominant source of genetic variation. In populations HSG, LCH, MPG, BPC, GX, and LD, between 50.0% and 70.0% of genotypes contributed to incompatibility and in each population the contribution of genotypes to incompatibility varied considerably. In particular, there was only a gradual decrease of conflict in the data set when genotypes with the highest incompatibility counts were successively deleted (Figure 2A–F). It was necessary to delete at least half the genotypes to obtain a tree-like structure in the data set, suggesting that sexual reproduction is more common in the populations and plays more essential roles in maintaining genetic variation than asexual reproduction [35], [36], [40]. This finding is in good agreement with other previous studies, which have nearly all used allozyme markers to infer the mating systems operating in natural populations of ferns. As summarized by Haufler [41]–[43], Soltis and Soltis [44], and Ranker and Geiger [9], allozyme studies showed that populations of diploid homosporous ferns are usually dominated by sexual random mating or outcrossing. Moreover, Chiou et al. [32] also observed that *A. spinulosa* produced sporophytes mainly by intergametophytic mating, especially intergametophytic crossing, by examining the sexuality of gametophytes in culture.

In contrast to the above-mentioned six populations, genetic variation in population ZHG appears to be primarily caused by somatic mutations. The incompatibility in its data set was quickly driven to zero upon deletion of only five of 15 (33.3%) genotypes; and the ten remaining genotypes exhibited a strictly tree-like data structure (Figure 2G). In addition, there were three genotypes detected to be over-represented in the population. This observed pattern of genetic variation can be most parsimoniously explained by acquisition of unique somatic mutations in clonal lineages [35], [40], [45], implying that asexual reproduction dominates in population ZHG. It had been noted quite early that sporophytes of the genus *Alsophila* have the ability to reproduce asexually both by apospory and induced proliferation [5]. Nevertheless, later studies insisted that tree ferns rarely reproduce asexually [24]. Here our molecular data provided further evidence for the natural occurrence of asexual reproduction in *A. spinulosa* (population ZHG). Population ZHG is located in Zi Huang Gou, Chishui, Guizhou, growing by streamsides under bamboo forest. Its habitat demonstrates no appreciable difference from that of other populations, especially HSG and LCH, which are also from Guizhou. However, our own survey, as well as Zhou et al.’s [33], found that population ZHG is more dense and continuous than other populations, and the number of individuals has been increasing during the last decades. The high proportion of asexual reproduction may be associated with the population expansion.

It has been predicted that asexual reproduction is expected to reduce population genetic variation and enhance genetic structure [11], [20]. Contrary to this, we detected no significant difference in genetic variation and differentiation between population ZHG, which is dominated by asexual reproduction, and other populations reproducing primarily by sexual reproduction (Table 3 and 5). It appears that the mode of reproduction had little effect on the genetic composition of population ZHG. This may be explained by the following reasons. First, some genetically divergent lineages of *A. spinulosa* have existed in population ZHG before the predominance of asexual reproduction, which account for most of the observed genetic variation [12]. Second, somatic mutations in clonal lineages can generate a considerable amount of genetic variation, as has been observed in the angiosperm *Saxifraga cernua* [40]. Third, a lack of admixture among the pre-existing lineages due to the later predominantly asexual reproduction would hamper the differentiation of population ZHG from other populations. Fourth, gene flow from the neighboring populations of HSG and LCH would slow the diversification but only via sexual reproduction.

Our results indicate that although sexual reproduction is predominant in most of the natural populations of *A. spinulosa*, asexual reproduction can indeed occur and dominate on a local scale. Similar patterns have been identified in a number of other fern species [8], [9], [10], [46]. The change of the primary mode of reproduction does not mediate significant difference in the population genetic composition. The present study clearly demonstrates how character compatibility analysis can contribute to separate the role of sexual and asexual components in shaping the genetic pattern of fern populations.

## Materials and Methods

### Ethics Statement

This study was conducted in accordance with all People’s Republic of China laws. No specific permits were required for the described field studies. No specific permissions were required for access to the locations or for activities described in this study. The location is not privately owned or protected in any way.

### Plant Materials

Ten natural populations of *Alsophila spinulosa* were sampled across its range in China. The locations are given in Figure 1. The code, geographical coordinates, and altitude of each population are listed in Table 2. Populations were defined as discrete groups of sporophytes located in the same zone without geographical barriers. Twenty individuals were selected randomly from each population at intervals of at least 10 m, giving a total of 140 individuals. Fresh frond material was collected directly from sporophytes using silica gel to dry and preserve samples.

### DNA Isolation

Total genomic DNA was isolated from ground frond tissue by means of the modified cetyltrimethylammonium bromide (CTAB) protocol [47]. DNA concentration and purity were determined by measuring ultraviolet (UV) absorption using a Pharmacia 2000 UV/Visible Spectrophotometer. The quantity and integrity of DNA samples were also assessed by 0.8% agarose gel electrophoresis.

### ISSR Amplification and Examination

PCR was performed in 20 µl reaction mixtures that contained 1 U *Taq* polymerase, 50 mM KCl, 10 mM Tris-HCl (pH 9.0), 0.1% Triton X-100, 2.0 mM MgCl_2_, 0.2 mM each dNTP, 0.3 µM primer, 80 ng genomic DNA, and DNA-free water. Amplifications were conducted in an MJ-Research PTC-100TM Peltier thermal cycler. Initial denaturation was at 94°C for 5 min, followed by 40 cycles of 94°C for 40 s, 53°C for 35 s, 72°C for 70 s, and a final elongation at 72°C for 5 min. A total of 80 ISSR primers (UBC ISSR Primers 802, 805, 807–881, 885, 886, and 888, University of British Columbia, Vancouver, Canada) were initially screened for amplification. Twenty primers that generated clear and reproducible banding patterns were chosen for the final ISSR analysis (Table 1). Negative controls were included in each experiment to check for the absence of contamination. For each sample, at least two amplifications were run to determine the reproducibility of the bands obtained. PCR products were separated using electrophoresis on 1.8% agarose gels stained with ethidium bromide, then digitally photographed under UV light.

### Data Analysis

ISSR banding patterns were scored in a binary form for band presence (1) or absence (0) and entered into a data matrix. The binary matrix was used to calculate the percentage of polymorphic loci, observed number of alleles, effective number of alleles, and Nei’s gene diversity [48] using POPGEN32 [49]. Shannon’s index of phenotypic diversity was also quantified using the equation *S*  =  −∑p_i_lnp_i_ where p_i_ is the frequency of a given band in the population [50]. Its 95% confidence interval was estimated by a sampled randomization test [51]. In addition, we used Mann-Whitney U-tests to examine differences in the estimates of genetic variation [52].

Holsinger et al. [53] developed a Bayesian approach that can provide unbiased estimates of allele frequency and population differentiation from banding data of dominant markers. Bayesian parameters *f* and *θ^B^* are analogues to the inbreeding coefficient (*F_IS_*) and the fixation index (*F_ST_*) of *F*-statistics [54], respectively. The posterior distributions of *f* and *θ^B^* were estimated numerically through Markov Chain Monte Carlo (MCMC) simulations by running HICKORY v1.0 [53]. The “*f*-free” model option was preferred due to its lower deviance information criterion and pD (measure of model complexity) values than for other models. Point estimates of *θ^B^*, as well as their 95% confidence intervals, were achieved with a burn-in of 50 000 iterations and a sampling run of 250 000 iterations from which every 50th sample was retained for posterior calculations. Three replicates were run to ensure that the results were consistent.

Character compatibility analysis was conducted to reveal the relative contribution of asexual and sexual reproduction in the population [33], [34]. In binary character data, such as the presence or absence of ISSR bands at two loci, the presence of all four character combinations (0/0, 1/0, 0/1, 1/1) is referred to as incompatibility, and will be more parsimoniously explained by recombination than by three mutation events. Thus, the sum of incompatible counts over all pairwise comparisons can serve as a measure of recombination [55]. In this study, the contribution of a particular genotype to matrix incompatibility was calculated by jackknifing using the JACTAX in PICA [55], and then the genotypes responsible for the greatest number of incompatibilities were successively deleted from the dataset until MIC reached zero. Asexual reproduction is expected to cause none or only a small number of recombinant genotypes that contribute to the overall matrix incompatibility [56], [57]; in such a case, there would be a sharp decrease of the incompatibility count upon deletion of these recombinant genotypes [34]. In contrast, if the deletion of nearly all genotypes is required to remove matrix incompatibility, the genotype differences should originate mostly from sexual recombination.
